# Glymphatic system impairment in cerebral small vessel disease: associations with perivascular space volume and cognition

**DOI:** 10.3389/fnagi.2025.1680094

**Published:** 2025-11-13

**Authors:** Lulu Ai, Zhiwei Li, Huipeng Huang, Chaojuan Huang, Shujian Chen, Xia Zhou, Xiaoqun Zhu, Zhongwu Sun

**Affiliations:** 1 Department of Neurology, The First Affiliated Hospital of Anhui Medical University, Hefei, China; 2 Department of Neurology, Mental and Neurological Disease Research Center, The Third Affiliated Hospital of Sun Yat-sen University, Guangzhou, China

**Keywords:** cerebral small vessel disease, cognitive impairment, diffusion tensor image analysis along the perivascular space, glymphatic function, subcortical perivascular space

## Abstract

**Objective:**

While emerging evidence links glymphatic dysfunction to cerebral small vessel disease (CSVD), its clinical relevance remains poorly defined. This study aimed to investigate the relationships among glymphatic function, perivascular space (PVS), and cognitive performance in CSVD.

**Methods:**

We enrolled 120 CSVD patients [52 with no cognitive impairment (CSVD-NCI) and 68 with mild cognitive impairment (CSVD-MCI)] and 40 healthy controls (HCs). Glymphatic function was assessed using the left ALPS index derived from diffusion tensor imaging analysis along the perivascular space (DTI-ALPS). Group comparisons in the ALPS index and PVS volume fraction (VF), and correlations among glymphatic function, PVS burden, and cognition were analyzed.

**Results:**

Compared to HCs, CSVD patients showed decreased ALPS index and increased PVS VF in basal ganglia (BG), caudate, putamen, and hippocampus, with more pronounced alterations in the left hemisphere. The ALPS index was inversely correlated with PVS VF in the BG (*r* = −0.232, *p* = 0.014), thalamus (*r* = −0.213, *p* = 0.024), caudate (*r* = −0.221, *p* = 0.019), and putamen (*r* = −0.210, *p* = 0.026) in CSVD. Furthermore, a lower ALPS index was associated with poorer performance in global cognition (*r* = 0.312, *p* = 0.001), executive function (*r* = 0.242, *p* = 0.012), processing speed (*r* = 0.264, *p* = 0.006), and visuospatial function (*r* = 0.272, *p* = 0.004). Finally, the ALPS index partially mediated the association between putamen-PVS VF and global cognitive function, especially in the left hemisphere.

**Conclusion:**

Our findings demonstrate that impaired glymphatic function was associated with enlarged BG-PVS, especially in the putamen, and worse cognitive performance, highlighting its potential role in disease progression and cognitive decline in CSVD.

## 1 Introduction

Cerebral small vessel disease (CSVD) is a progressive cerebrovascular disease characterized by diverse clinical manifestations, especially neurocognitive dysfunction, a primary contributor to vascular cognitive impairment (Wardlaw et al., 2019; Duering et al., 2023). The high prevalence and substantial severity of cognitive decline impose a significant socioeconomic burden on CSVD patients. The glymphatic system, a brain waste clearance system first described by Iliff et al. (2012), facilitates the exchange of cerebrospinal fluid (CSF) with interstitial fluid (ISF). In this process, CSF enters the brain parenchyma via para-arterial perivascular spaces, passes through astrocytic aquaporin-4 (AQP4) water channels, and mixes with ISF before being cleared along perivenous routes, thereby promoting the removal of metabolic waste (Mestre et al., 2020).

Recent evidence suggests that impaired glymphatic function may represent a final common pathway in the pathogenesis of dementia (Nedergaard and Goldman, 2020) and has been increasingly implicated in the pathophysiology of CSVD (Tang et al., 2022; Tian et al., 2022; Tian et al., 2023; Yang et al., 2025). However, the relationship between glymphatic dysfunction and CSVD is likely bidirectional and multifactorial. On one hand, CSVD-related pathologies, such as endothelial dysfunction, blood–brain barrier disruption, and reduced arterial pulsatility, may impair glymphatic flow by compromising perivascular pumping mechanisms and fluid transport (Mestre et al., 2018; Nedergaard and Goldman, 2020). On the other hand, impaired glymphatic clearance may exacerbate CSVD by allowing the accumulation of neurotoxic waste products, such as amyloid-*β* and tau proteins, and pro-inflammatory molecules within perivascular spaces, further damaging vascular integrity and promoting white matter injury (Wardlaw et al., 2020; Zhang et al., 2021). This vicious cycle may accelerate cognitive decline and structural brain damage, positioning glymphatic dysfunction as both a consequence and a driver of CSVD progression.

The assessment of glymphatic function *in vivo* remains a methodological challenge. Intravital imaging techniques, such as in vivo two-photon microscopy with fluorescent tracers injected into the CSF, are considered the benchmark for directly visualizing CSF-ISF exchange and solute clearance (Iliff et al., 2012). However, these invasive approaches are largely confined to animal studies. In humans, dynamic contrast-enhanced MRI (DCE-MRI) using gadolinium-based contrast agents (GBCA) has been widely used as an indirect method to evaluate glymphatic-related solute clearance (Ringstad et al., 2017). Nevertheless, its invasive nature and safety concerns limit its broad application. While intravenous administration of GBCA represents a routine clinical procedure, its necessity in observational research contexts, particularly in conditions like CSVD, where repeated scanning might be desirable, raises practical and ethical considerations. These include the small yet non-negligible risk of adverse reactions, the potential for gadolinium deposition in tissues, and the necessary exclusion of individuals with contraindications, such as renal impairment. Consequently, the development of non-invasive alternatives remains crucial to enhance applicability and enable repeated assessments across the disease course. The diffusion tensor imaging analysis along the perivascular space (DTI-ALPS) index provides such a non-invasive alternative (Taoka et al., 2017). The method utilizes routinely acquired diffusion tensor imaging data without requiring contrast administration, thereby eliminating associated risks and broadening the eligible participant population. This approach holds particular relevance for investigating CSVD, a chronic and progressive disorder in which serial assessment of glymphatic function may offer critical insights into disease trajectory. Furthermore, the ALPS index specifically quantifies water diffusion along perivascular spaces, a process conceptually linked to glymphatic convective flow, and has shown strong correlation with gadolinium-based glymphatic MRI evaluations (Zhang et al., 2021; Liu et al., 2024). For these reasons, we employed the DTI-ALPS method to assess glymphatic function in our cohort.

Perivascular spaces (PVS), fluid-filled spaces surrounding cerebral small vessels, play an essential role in brain waste clearance. The enlargement of PVS (ePVS), thought to reflect impaired fluid dynamics and waste accumulation, is increasingly recognized as a key feature of CSVD pathophysiology (Wardlaw et al., 2020; Mestre et al., 2017; Brown et al., 2018). Although numerous studies have reported associations between ePVS and dementia (Ding et al., 2017; Romero et al., 2022; Zhang et al., 2024), others have failed to replicate this link (Benjamin et al., 2018; Hilal et al., 2018; Gertje et al., 2021). A major methodological limitation in PVS research has been the reliance on semi-quantitative visual rating scales, which are time-consuming and error-prone (Doubal et al., 2010). To address this, we implemented an Enhanced PVS Contrast (EPC) pipeline combined with a dedicated filtering algorithm to improve PVS visibility and enable volumetric quantification of PVS burden (Sepehrband et al., 2019).

While numerous studies have documented enlarged PVS in CSVD (Wang J. et al., 2023; Wang M. et al., 2023), direct *in vivo* evidence linking this structural alteration to impaired glymphatic function remains limited. Therefore, this study aimed to: (1) quantify differences in the ALPS index and PVS volume fraction between CSVD patients and healthy controls and (2) examine the interrelationships among glymphatic function (as measured by the ALPS index), PVS volume, and cognitive performance in CSVD.

## 2 Materials and methods

### 2.1 Participants

In this study, a total of 120 CSVD participants, including 52 with no cognitive impairment (CSVD-NCI) and 68 with mild cognitive impairment (CSVD-MCI), were included. Inclusion criteria included: (1) age 50–80 years; (2) native Chinese speakers and right-handed; and (3) MRI meeting the imaging standards for CSVD diagnosis (Wardlaw et al., 2013). The total CSVD burden was assessed using Wardlaw’s scale (0–4 score), with a score of ≥1 indicating the presence of CSVD. Exclusion criteria included: (1) significant intracranial pathologies that could confound imaging assessments, including space-occupying lesions (e.g., brain tumors or abscesses), non-CSVD related structural abnormalities (e.g., post-traumatic encephalomalacia, cerebral malformations, or hydrocephalus), and other disorders affecting white matter integrity or cognitive function (e.g., demyelinating plaques suggestive of multiple sclerosis or sequelae of extensive encephalitis); (2) major cerebrovascular diseases distinct from CSVD, specifically cortical or large subcortical infarcts attributable to large-artery atherosclerosis or cardiogenic embolism; (3) clinically significant psychiatric or consciousness-impairing conditions; (4) history of malignant tumor; (5) severe cardiovascular, liver, or kidney dysfunction; (6) inability to complete cognitive assessment or MRI scanning.

During the same period, 40 healthy controls (HCs) were included, who did not meet the inclusion criteria for the CSVD group. All the participants provided written informed consent in accordance with the Declaration of Helsinki, and the Human Subjects Review Committee of the First Affiliated Hospital of Anhui Medical University approved this study (approval number: PJ2023-01-45).

### 2.2 Baseline clinical characteristics and classification of cognitive function

Demographic characteristics, including age, sex, and years of education, were recorded for each subject by a trained neurologist. The vascular risk factors (VRFs) included hypertension, diabetes, hypercholesterolemia, smoking, and body mass index (BMI) using a standardized questionnaire (van Norden et al., 2011; Gottesman et al., 2017). Patients with CSVD were divided into two groups according to the clinical dementia rating (CDR) score (Gorelick et al., 2011): participants with mild cognitive impairment (CDR = 0.5, CSVD-MCI group) and participants with no cognitive impairment (CDR = 0, CSVD-NCI group).

### 2.3 Neuropsychological assessment

All participants underwent a neuropsychological assessment in a quiet, comfortable environment within a week of their MRI examination, conducted by an experienced neurologist. The Chinese Montreal Cognitive Assessment (MoCA) was employed to assess the global cognitive function (Huang et al., 2018). To provide a more nuanced analysis beyond global screening, specific cognitive domains were assessed with a comprehensive test battery, specifically: memory function [Auditory Verbal Learning Test (AVLT); Feng et al., 2021], executive function [Trail Making Test-B (TMT-B)], processing speed [Trail Making Test-A (TMT-A); Tombaugh, 2004], visuospatial function [10-point clock drawing test (CDT-10); Hshieh et al., 2018], and language function [Boston Naming Test (BNT); Williams et al., 1989]. The raw scores from each cognitive test were converted to z-scores, standardized to the mean and standard deviation of the healthy control group in this cohort (z-score = individual test score minus the means of HCs, divided by the standard deviation of HCs) (Wang J. et al., 2023; Wang M. et al., 2023). Furthermore, in all subsequent statistical models where these z-scores served as dependent variables, we explicitly included age and years of education as covariates. Notably, the original scores of the TMT were based on the completion time; therefore, the z-scores of the processing speed and executive function were inverted by multiplying them by −1, with higher z-scores representing better performance (Huang et al., 2020).

### 2.4 MRI protocols

We acquired MRI data using a 3.0-Tesla MR system (Discovery MR750w, General Electric, Milwaukee, WI, USA) with a 24-channel head coil. To reduce scanning noise, participants wore earplugs and foam padding restricted head movement. Simultaneously, participants were instructed to remain awake with eyes closed in a relaxed state. High resolution three-dimensional T1-weighted (3D-T1) images were acquired through a brain volume (BRAVO) sequence with the following parameters: slice thickness = 1.0 mm, repetition time (TR) = 8.464 ms, echo time (TE) = 3.248 ms, inversion time (TI) = 450 ms, flip angle (FA) = 12°, field of view (FOV) = 256 mm × 256 mm, matrix size = 256 × 256, slice thickness = 1 mm without gap, 188 sagittal slices, and acquisition time = 296 s. T2 fluid-attenuated inversion recovery (T2 FLAIR) images were acquired with the following parameters: TR = 9,000 ms, TE = 119.84 ms, FA = 160°, FOV = 225 mm × 225 mm, matrix size = 512 × 512, number of layers = 19, layer thickness = 7 mm, and acquisition time = 1 min 57 s. Susceptibility-weighted imaging (SWI) images were acquired with the following parameters: TR = 45.4 ms, TE = 23.536 ms, FA = 20°, FOV = 240 mm × 240 mm, matrix size = 512 × 512, slice thickness = 1 mm, number of slices = 138, and acquisition time = 3 min 51 s. Diffusion tensor imaging (DTI) was conducted using a spin echo single-shot echo planar imaging (SE-SS-EPI) sequence with the following parameters: slice thickness = 3 mm without gap, TR = 10,000 ms, TE = 74.2 ms, FOV = 256 mm × 256 mm, matrix size = 128 × 128, FA = 90°, 50 axial slices, 64 diffusion gradient directions (b = 1,000 s/mm2) plus five b = 0 reference images, and acquisition time = 700 s. Routine T2-weighted images were also collected to exclude any organic brain abnormality.

### 2.5 Quantification of DTI-ALPS index

**Figure 1:**
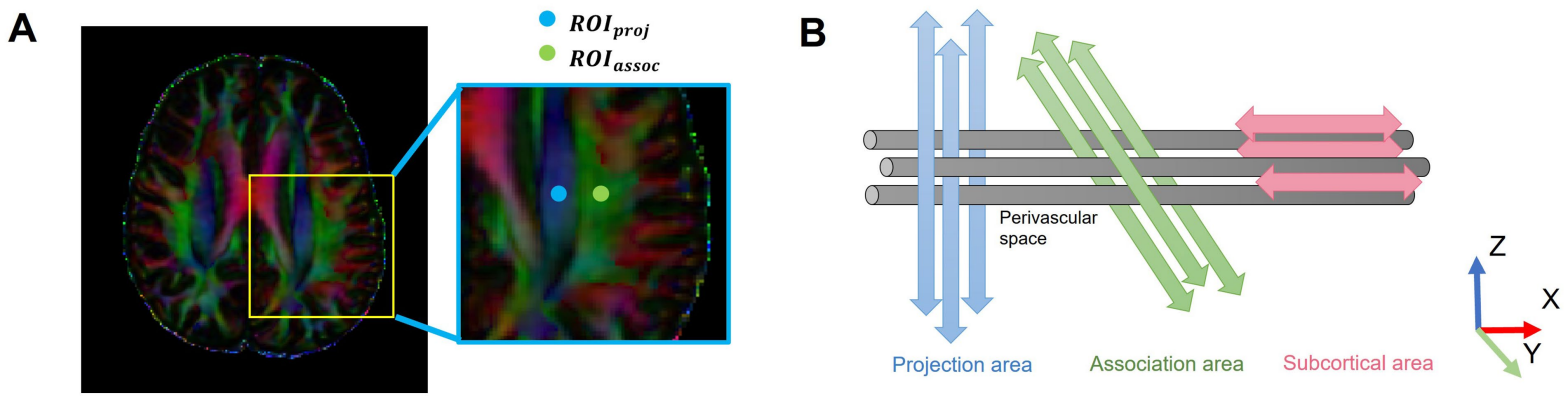
Schematic of the DTI-ALPS methodology. **(A)** Two ROIs were placed on the projection fibers (blue; *z*-axis) and association fibers (green; *y*-axis) in the left hemisphere, where the deep medullary veins were vertical to the ventricular body, on a color-coded FA map. **(B)** The schematic diagram shows the relationship between the direction of the PVS (gray cylinders) and subcortical fibers (red; *x*-axis), association fibers (green; *y*-axis), and projection fibers (blue; *z*-axis). The direction of the PVS is perpendicular to projection and association fibers. DTI-ALPS, diffusion tensor imaging analysis along the perivascular space; ROIs, regions of interest; PVS, perivascular spaces; proj, projection fibers; assoc., association fibers.

DTI-ALPS calculation was performed in accordance with previous studies. DTI images were processed using FMRIB’s Diffusion Toolbox of the FMRIB Software Library V.6.0 (FSL, http://www.fmrib.ox.ac.uk/fsl/), including non-brain tissues removal, head motion, eddy current correction, and DTI parameter calculation (fractional anisotropy [FA] and color-coded FA map). All regions of interest (ROIs) were independently placed by two trained raters (LL.A. and X.Z.) [both with >3 years of experience in neuroimaging analysis and trained under the supervision of a senior neuroradiologist (JJ.Z.)], both of whom were blinded to participant group assignment and cognitive status. The raters adhered to a standardized protocol. On the color-coded FA map, two 5-mm diameter spherical ROIs were placed at the level of the lateral ventricle body where the deep medullary veins are perpendicular to the ventricle. The center of the ROI for the projection fibers was placed on the blue-colored area, and the center for the association fibers was placed on the green-colored area (Figure 1). Discrepancies in ROI placement between raters were resolved through consensus with the senior neuroradiologist (J.J.Z.). Inter- and intra-rater reliability were assessed in a randomly selected subset of 30 participants using the intraclass correlation coefficient (ICC) based on a two-way mixed-effects model for absolute agreement (Koo and Li, 2016). Excellent reliability was observed, with an inter-rater ICC of 0.94 (95% CI, 0.89–0.97) and an intra-rater ICC of 0.96 (95% CI, 0.92–0.98).

The ALPS index was calculated only in the left hemisphere because all participants were right-handed, making the left hemisphere dominant with more developed association fibers relevant to cognition and thus a more representative measurement site. Furthermore, unilateral assessment is a common practice in the DTI-ALPS field, which helps avoid confounding factors introduced by inter-hemispheric variability, ensures methodological consistency, and aligns with established protocols (Tian et al., 2023; Zhang et al., 2021; Taoka et al., 2017). Diffusivities in the directions of the *x*-axis (*Dxx*), *y*-axis (*Dyy*), and *z*-axis (*Dzz*) of each ROI were recorded. The equation for the ALPS index is as follows:


$$\text{ALPS index}=\frac{\text{mean}(\text{Dxxproj},\text{Dxxassoc})}{\text{mean}(\text{Dyyproj},\text{Dzzassoc})}$$

### 2.6 Quantitative measurement of PVS volume fraction

PVS mapping was conducted using an Enhanced PVS Contrast (EPC) multimodal approach, developed by Sepehrband et al. (2019), which enhances PVS visibility on MRI by fusing T1- and T2-weighted images and removing non-structured high-frequency spatial noise with a dedicated filtering algorithm. Preprocessing involved non-uniform intensity normalization, intensity normalization, and skull stripping. Then, FLAIR images were corrected for non-uniform field inhomogeneities using advanced normalization tools (ANTs) and co-registered to Montreal Neurological Institute (MNI)-152 space using FSL’s FNIRT through linear and non-linear transformations. To enable precise PVS quantification, subcortical nuclei masks were first automatically segmented from the T1-weighted images using FSL’s FIRST tool. The analyzed regions included the basal ganglia and their subdivisions (amygdala, caudate, putamen, pallidum), along with the thalamus and hippocampus. Subsequently, whole-brain tissue segmentation into gray matter, white matter, and cerebrospinal fluid was conducted using FSL’s FAST tool. Then, T1w and T2w images were filtered using an adaptive non-local mean filtering technique, and EPC was obtained by dividing filtered images (i.e., T1w/T2w) (Manjón et al., 2010). Last, the Frangi filter provided by the Quantitative Imaging Toolkit (QIT) was used to generate a quantitative map of vesselness in the regions of interest with default parameters.

PVS volumes were automatically computed by summing delineated PVS volumes in each region (Benjamin et al., 2018). The PVS volume fraction was calculated as the ratio of PVS volume to the sum of gray matter and white matter volumes, eliminating effects of interindividual brain size variability. Given the well-established role of deep subcortical PVS as a core neuroimaging marker of CSVD and its strategic location along the glymphatic pathway (Wardlaw et al., 2020; Brown et al., 2018), our quantitative analysis specifically targeted subcortical nuclei. A complete list of all analyzed regions and their results are provided in Supplementary Table S1. The equation for PVS volume fraction is as follows:


$$\mathrm{PVS}\;\text{volume faction}\;(\mathrm{PVS}\;\mathrm{VF})=\frac{\;\mathrm{PVS}\;\text{volume}}{\text{gray}+\text{white matter volume}}\times 100\%$$

### 2.7 Statistical analysis

Statistical analysis was performed using R software (version 4.1.1) and IBM SPSS Statistics (version 26). The normality of continuous variables was assessed using the Shapiro–Wilk test, and the homogeneity of variances was verified using Levene’s test. Based on these assessments, normally distributed variables were expressed as mean ± standard deviation (Mean ± SD) and compared using one-way analysis of variance (ANOVA), while non-normally distributed variables were expressed as median (interquartile range, IQR) and analyzed using the Kruskal–Wallis test. Categorical variables were presented as counts (percentages, %) and assessed with a Chi-square test. Group comparisons of ALPS index and PVS volume fraction were performed with Bonferroni correction, controlling for age and sex (corrected *p* < 0.05).

Partial correlation analysis between the ALPS index and PVS VF was adjusted for age, sex, years of education, and VRFs (hypertension, diabetes, hypercholesterolemia, smoking, and BMI). Partial correlation analysis between ALPS index and cognitive function was adjusted for age, sex, years of education, VRFs (hypertension, diabetes, hypercholesterolemia, smoking, and BMI), and neuroimaging markers of CSVD (WMH Fazekas scores, presence of lacunes, presence of CMBs, and BG-ePVS grade). The mediation effects of the ALPS index on the association between PVS VF and cognition were further assessed with age, sex, years of education, and VRFs (hypertension, diabetes, hypercholesterolemia, smoking, and BMI) as covariates. PROCESS macro^1^ software was used for mediation analysis. Based on 5,000 bootstrap realizations, a significant indirect effect was determined if the bootstrap 95% confidence interval (CI) excluded zero. Statistical significance was set at *p* < 0.05, with false discovery rate (FDR) correction for multiple correlation tests.

## 3 Results

### 3.1 Demographic, neuropsychological, and neuroimaging characteristics

Demographic, neuropsychological, and neuroimaging characteristics are summarized in Table 1. The study included 120 CSVD patients (61 men and 59 women, mean age 63.69 ± 6.84 years) and 40 HC (19 men and 21 women, mean age 60.18 ± 4.57 years). No significant differences were found in sex, education, smoking, diabetes, hypercholesterolemia, or BMI among the three groups. The CSVD group, particularly the CSVD-MCI subgroup, was older than the HCs. Both CSVD subgroups showed a higher prevalence of hypertension compared to HCs. The CSVD-MCI group exhibited worse cognitive performance compared to the CSVD-NCI and HC groups across all assessed domains. Both CSVD-MCI and CSVD-NCI groups differed significantly from HCs in all neuroimaging markers, with the CSVD-MCI group demonstrating higher WMH Fazekas scores and greater BG-ePVS (basal ganglia-ePVS) than the CSVD-NCI group.

**Table 1:** Baseline demographic, cognitive function, and neuroimaging characteristics among HC, CSVD-NCI, and CSVD-MCI groups.

Variables	HC (*n* = 40)	CSVD (*n* = 120)	*p* value	CSVD-NCI (*n* = 52)	CSVD-MCI (*n* = 68)	*p* value
Demographics						
Age, years	60.18 ± 4.57	63.69 ± 6.84	<0.001	62.38 ± 6.96	64.69 ± 6.62	0.0017^‡^
Male, *n* (%)	19 (47.50)	61 (50.83)	0.7150	28 (53.85)	33 (48.53)	0.7919
Education, years	9.43 ± 4.10	8.53 ± 3.84	0.2106	9.09 ± 3.85	8.10 ± 3.80	0.1797
Smoking, *n* (%)	5 (12.50)	37 (30.83)	0.0225	16 (30.77)	21 (30.88)	0.0740
Hypertension, *n* (%)	14 (35.00)	78 (65.00)	<0.001	33 (63.46)	45 (66.18)	0.0038^†,‡^
Diabetes, *n* (%)	6 (15.00)	21 (17.50)	0.7147	9 (17.31)	12 (17.65)	0.9342
Hypercholesterolemia, *n* (%)	10 (25.00)	34 (28.33)	0.6826	15 (28.85)	19 (27.94)	0.9143
BMI (kg/m^2)	23.77 ± 2.28	23.84 ± 2.81	0.8789	23.78 ± 2.55	23.89 ± 3.02	0.9655
Neuropsychological assessments						
MoCA total scores	25.98 ± 1.86	22.18 ± 4.23	<0.001	25.25 ± 2.29	19.82 ± 3.85	<0.001^‡,§^
CDR scores	0.00 ± 0.00	0.28 ± 0.25	<0.001	0.00 ± 0.00	0.49 ± 0.09	<0.001^‡,§^
Memory function	0.00 ± 1.00	−0.75 ± 1.37	0.0017	−0.21 ± 1.01	−1.16 ± 1.46	<0.001^‡,§^
Executive function	0.00 ± 1.00	−1.58 ± 2.64	<0.001	−0.59 ± 1.92	−2.33 ± 2.87	<0.001^‡,§^
Processing speed	0.00 ± 1.00	−1.02 ± 2.14	<0.001	−0.34 ± 1.39	−1.54 ± 2.45	<0.001^‡,§^
Language function	0.00 ± 2.92	−1.38 ± 3.52	0.0269	−0.09 ± 3.26	−2.37 ± 3.41	<0.001^‡,§^
Visuospatial function	0.00 ± 1.00	−2.50 ± 4.09	<0.001	−0.29 ± 1.14	−4.19 ± 4.69	<0.001^‡,§^
Neuroimaging markers						
WMH Fazekas scores	1.00 (0.00, 2.00)	4.00 (3.00, 6.00)	<0.001	4.00 (3.00, 5.00)	5.00 (4.00, 6.00)	<0.001^†,‡,§^
Lacunes, *n* (%)	0 (0.00)	30 (25.00)	<0.001	14 (26.92)	16 (23.53)	0.0019^†,‡^
CMBs, *n* (%)	0 (0.00)	55 (45.83)	<0.001	22 (42.31)	33 (48.53)	<0.001^†,‡^
BG-ePVS, *n* (%)	0 (0.00)	73 (60.83)	<0.001	25 (48.08)	48 (70.59)	<0.001^†,‡,§^
CSVD total burden	0.00 (0.00, 0.00)	2.00 (1.00, 3.00)	<0.001	2.00 (1.00, 3.00)	2.00 (2.00, 3.00)	<0.001^†,‡^

Data are presented as means ± standard deviation for the continuous variables and as frequency (percentage) for the categorical variables. Normally distributed continuous variables were compared using one-way ANOVA (with post-hoc Bonferroni tests for pairwise comparisons); non-normally distributed continuous variables were compared using the Kruskal-Wallis test (with post-hoc Bonferroni-corrected Mann–Whitney U tests for pairwise comparisons); Categorical variables were compared using the Chi-square test. Cognitive domain z-scores were derived from the following neuropsychological tests: Memory function: Auditory Verbal Learning Test (AVLT); Executive Function: Trail Making Test-B (TMT-B); Processing Speed: Trail Making Test-A (TMT-A); Language function: Boston Naming Test (BNT); Visuospatial Function: Clock Drawing Test (CDT-10).

HC, healthy control; CSVD, cerebral small vessel disease; NCI, no cognitive impairment; MCI, mild cognitive impairment; BMI, body mass index; MoCA, Montreal Cognitive Assessment; CDR, Clinical Dementia Rating; WMH, white matter hyperintensity; CMBs, microbleeds; BG, basal ganglia; ePVS, enlarged perivascular space.

^†^CSVD-NCI significantly different from HC. ^‡^CSVD-MCI significantly different from HC. ^§^CSVD-MCI significantly different from CSVD-NCI. *p* values of pairwise comparisons using Bonferroni correction.

### 3.2 Comparisons of the ALPS index among different diagnostic groups

**Figure 2:**
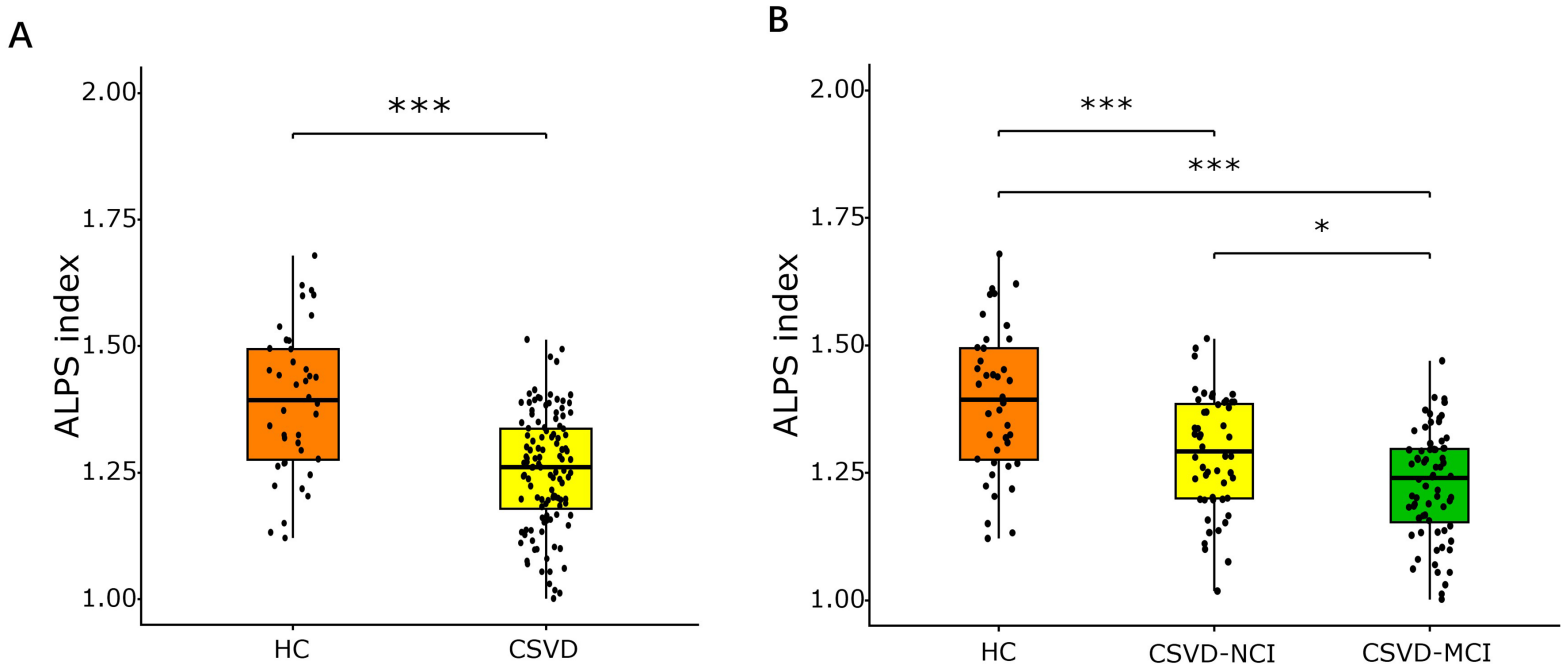
Box plots of comparison of ALPS index among different diagnostic groups. **(A)** Comparison of the ALPS index between HC and CSVD groups, assessed using an independent samples *t*-test. **(B)** Comparison of ALPS index in HC, CSVD-NCI, and CSVD-MCI groups, assessed using one-way ANOVA with post-hoc Bonferroni correction. ALPS, analysis along the perivascular space; HC, healthy control; CSVD, cerebral small vessel disease; NCI, no cognitive impairment; MCI, mild cognitive impairment. *p < 0.05, **p < 0.01, ***p < 0.001 after Bonferroni correction.

The mean ALPS index was 1.39 ± 0.14, 1.29 ± 0.11, and 1.23 ± 0.11 in HCs, CSVD-NCI, and CSVD-MCI groups, respectively (Supplementary Table S1). Compared to HCs, the CSVD group showed a significantly lower ALPS index (*p* < 0.001, Figure 2A). Post-hoc comparisons revealed a graded reduction across groups: the CSVD-MCI group exhibited a lower ALPS index than both the CSVD-NCI group and HCs, and the CSVD-NCI group had a lower ALPS index than HCs (all *p* < 0.05, Figure 2B).

### 3.3 Comparisons of the PVS VF among different diagnostic groups

**Figure 3:**
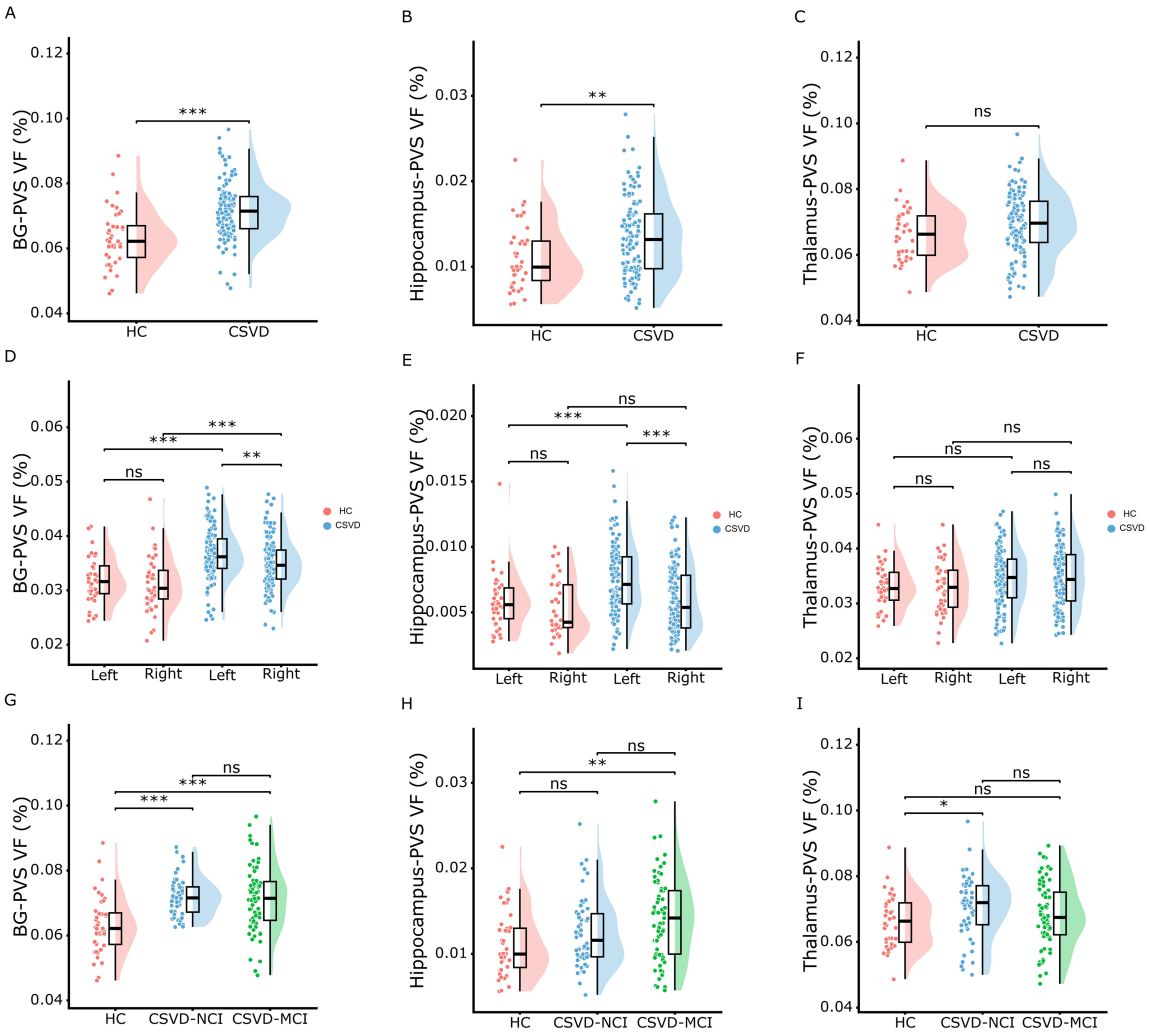
Rain-cloud plots of comparison of the subcortical nucleus PVS VF among different diagnostic groups. **(A–C)** Comparisons of the total PVS VF in BG, hippocampus, and thalamus between HC and CSVD groups. **(D–F)** Comparisons of the PVS VF in left and right hemispheres between HC and CSVD groups in BG, hippocampus, and thalamus. **(G–I)** Comparisons of the total PVS VF in BG, hippocampus, and thalamus among HC, CSVD-NCI, and CSVD-MCI groups. Group differences for normally distributed PVS VF data were assessed using one-way ANOVA with post-hoc Bonferroni correction. PVS VF, perivascular space volume fraction; HC, healthy control; CSVD, cerebral small vessel disease; NCI, no cognitive impairment; MCI, mild cognitive impairment; BG, basal ganglia. *p < 0.05, **p < 0.01, ***p < 0.001 after Bonferroni correction.

**Figure 4:**
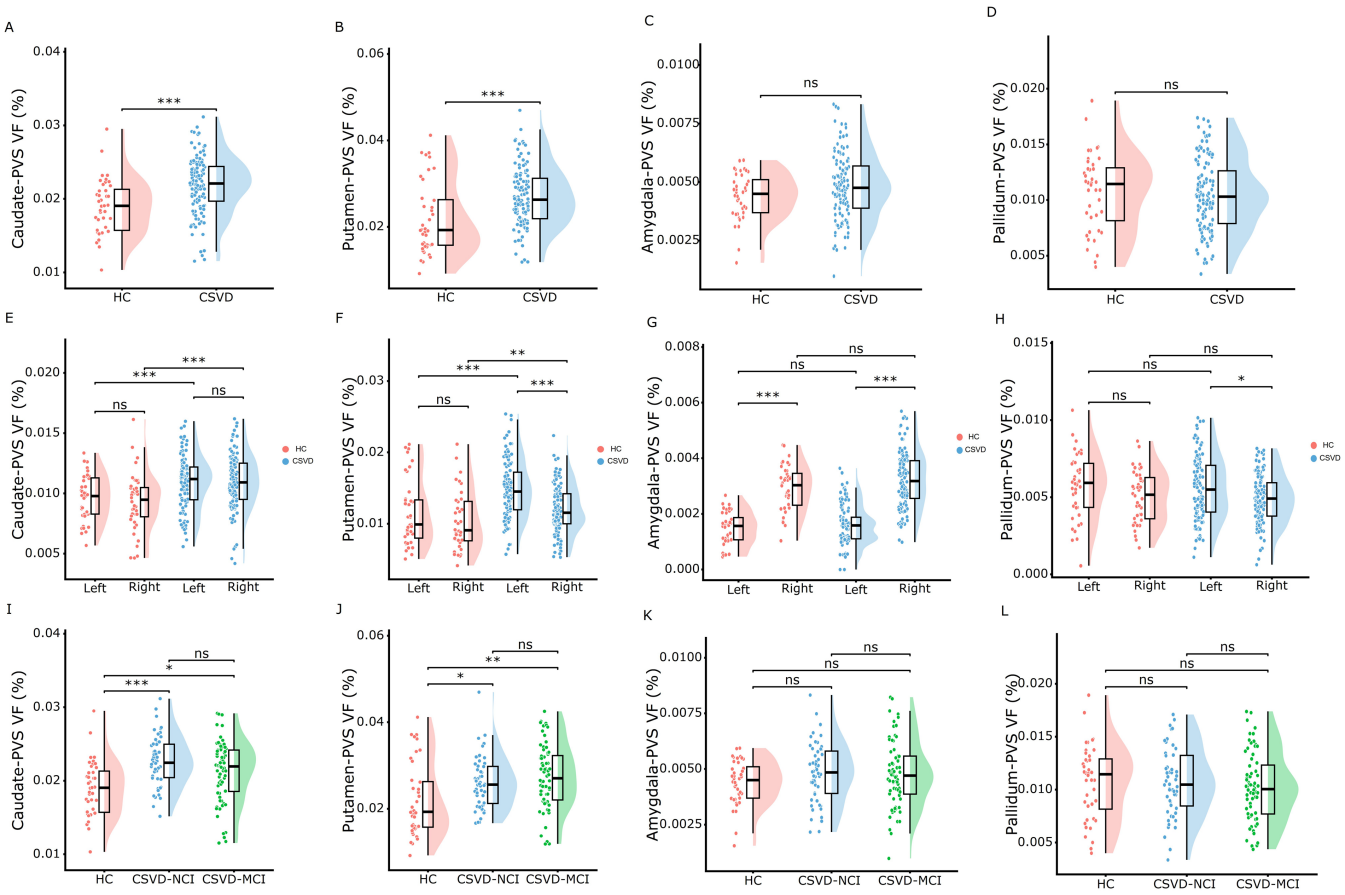
Rain-cloud plots of comparison of the PVS VF among BG subdivisions across diagnostic groups. **(A–D)** Comparisons of the total PVS VF in caudate, putamen, amygdala, and pallidum between HC and CSVD groups. **(E–H)** Comparisons of the PVS VF in left and right hemispheres between HC and CSVD groups in caudate, putamen, amygdala, and pallidum. **(I–L)** Comparisons of the total PVS VF in caudate, putamen, amygdala, and pallidum among HC, CSVD-NCI, and CSVD-MCI groups. Group differences for normally distributed PVS VF data were assessed using one-way ANOVA with post-hoc Bonferroni correction. PVS VF, perivascular space volume fraction; HC, healthy control; CSVD, cerebral small vessel disease; NCI, no cognitive impairment; MCI, mild cognitive impairment; BG, basal ganglia. *p < 0.05, **p < 0.01, ***p < 0.001 after Bonferroni correction.

As shown in Figure 3 and Supplementary Table S1, the CSVD group and both CSVD subgroups showed significantly higher PVS volume fraction in the bilateral BG compared to HCs (Figures 3A,D), with no statistical differences between CSVD-NCI and CSVD-MCI groups (Figure 3G). A detailed subregional analysis of BG revealed a similar change pattern in the caudate (Figures 4A,E,I) and putamen (Figures 4B,F,J), presented in Figure 4. In contrast, there were no group differences in PVS VF within the amygdala (Figures 4C,G,K) or pallidum (Figures 4D,H,L). Notably, within the CSVD group, the left BG (Figure 3D) and putamen (Figure 4F) exhibited significantly higher PVS VF than the right hemisphere.

The CSVD-MCI group exhibited higher hippocampus-PVS VF compared to both HCs and the CSVD-NCI group, whereas no difference was observed between the CSVD-NCI and HC groups (Figures 3B,E,H). Thalamus-PVS VF did not differ significantly among the three groups (Figures 3C,F,I).

### 3.4 Correlation analysis between ALPS index and PVS VF in CSVD group

**Figure 5:**
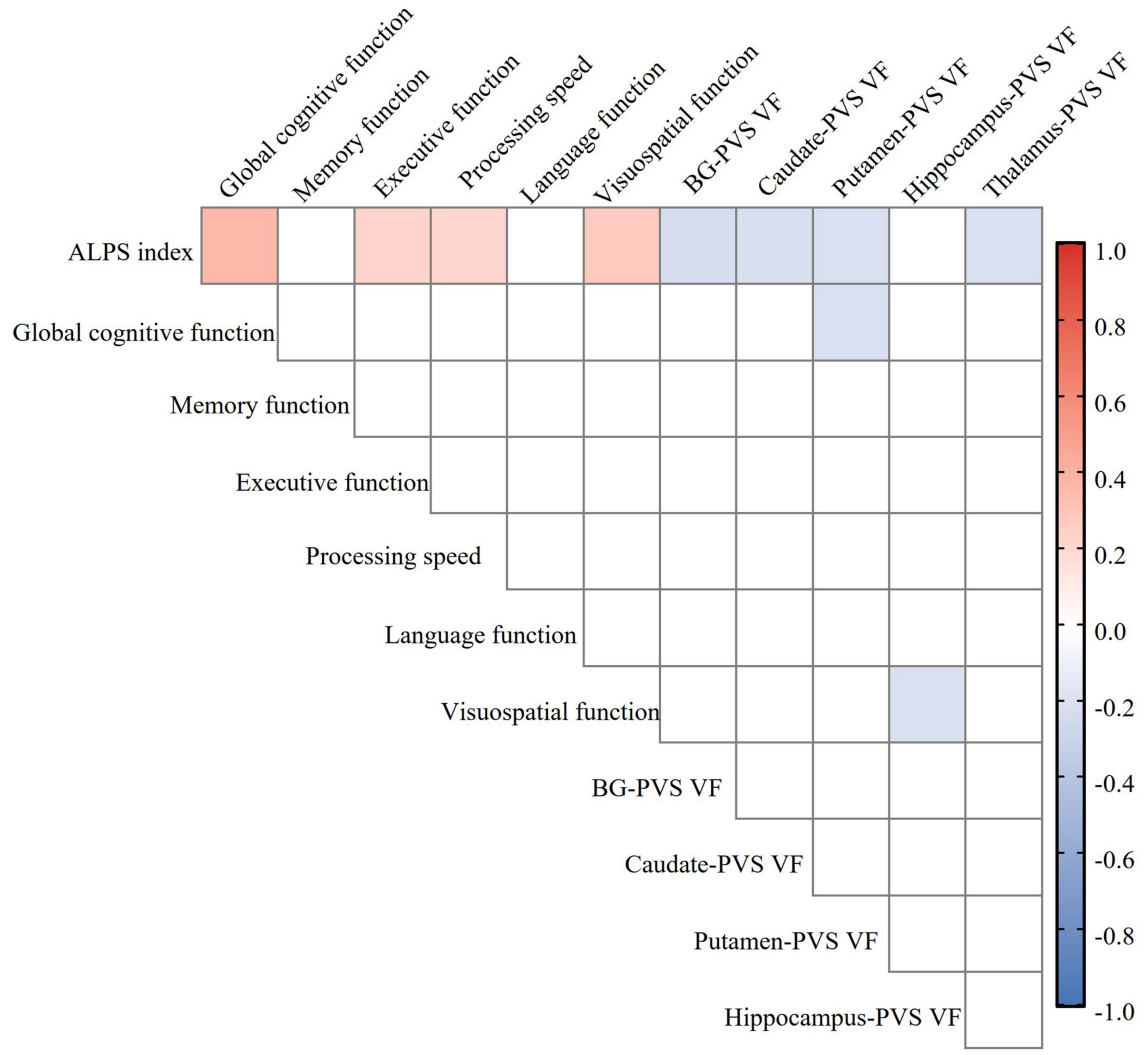
Heatmap of the relationship between ALPS index, perivascular space volume fraction (PVS VF), and cognitive function in the CSVD group. Color coding is employed to visually indicate the strength and direction of the correlation: blue for negative correlation, red for positive correlation, with deeper colors indicating stronger correlations (FDR correction, *p* < 0.05). ALPS, analysis along the perivascular space; PVS VF, perivascular space volume fraction; CSVD, cerebral small vessel disease; BG, basal ganglia; FDR, false discovery rate.

Partial correlation analysis revealed a significant negative correlation between ALPS index and BG-PVS VF (*r* = −0.232, *p* = 0.014), thalamus-PVS VF (*r* = −0.213, *p* = 0.024), caudate-PVS VF (*r* = −0.221, *p* = 0.019), and putamen-PVS VF (*r* = −0.210, *p* = 0.026) in the CSVD group, with no correlation in hippocampus-PVS, amygdala-PVS, or pallidum-PVS (Figure 5). These correlations remained statistically significant after FDR correction for multiple comparisons. Furthermore, the ALPS index was significantly negatively associated with WMH Fazekas scores, BG-ePVS, and CSVD total burden, but not with the presence of lacunes or CMBs. The complete results of the partial correlation analyses are available in Supplementary Table S2.

### 3.5 Correlation analysis between the ALPS index and cognitive function in the CSVD group

Partial correlation analysis, controlling for age, sex, years of education, VRFs, and neuroimaging markers of CSVD, demonstrated significant positive correlations between ALPS index and global cognitive function (*r* = 0.312, *p* = 0.001), executive function (*r* = 0.242, *p* = 0.012), processing speed (*r* = 0.264, *p* = 0.006), and visuospatial function (*r* = 0.272, *p* = 0.004), while there was no correlation in memory or language function in the CSVD group (Figure 5). These correlations remained statistically significant after FDR correction for multiple comparisons. The complete results of the partial correlation analyses are available in Supplementary Table S3.

### 3.6 Mediation analysis among PVS VF, ALPS index, and cognition in CSVD group

**Figure 6:**
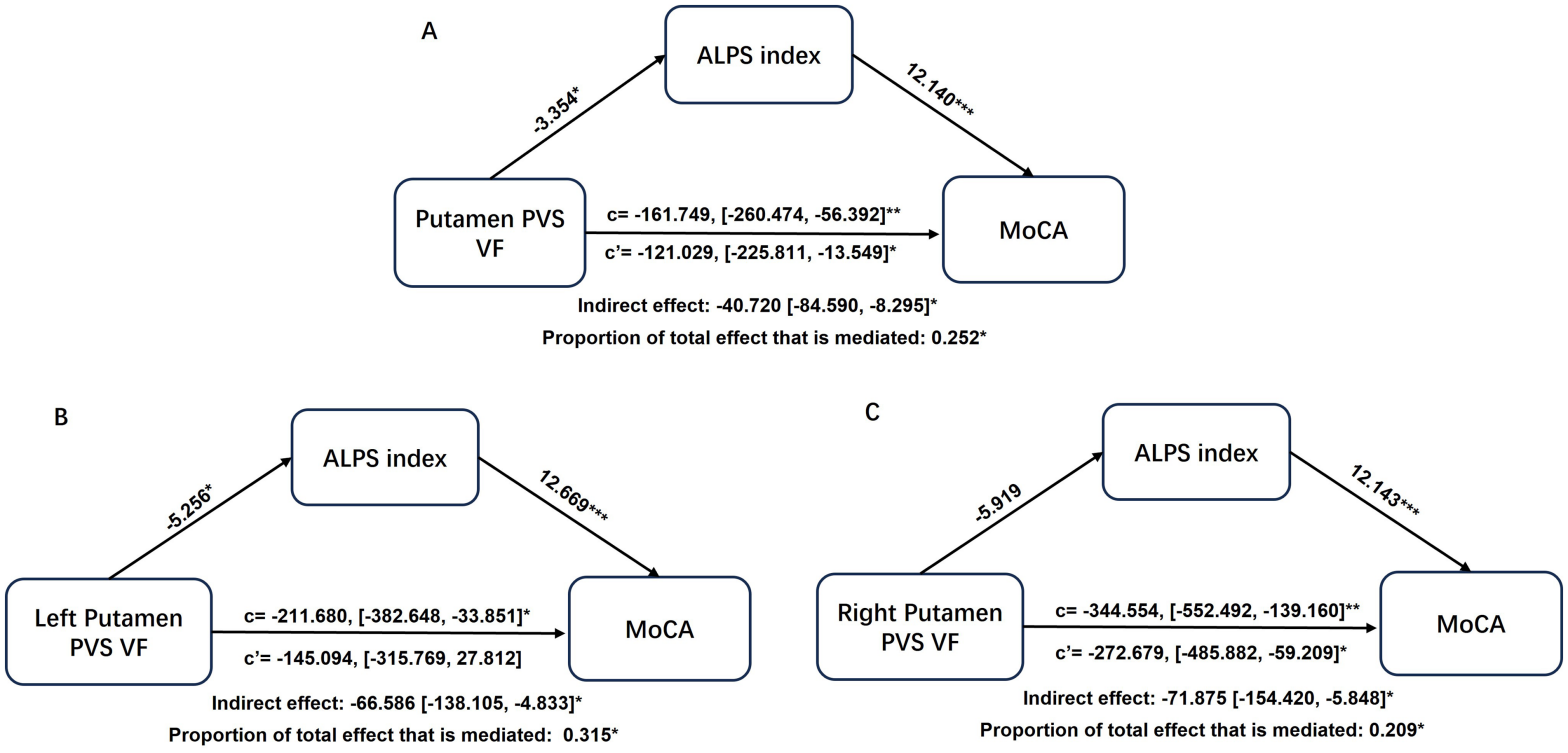
Mediation analysis of ALPS index in the associations of putamen-PVS VF **(A)**, left putamen-PVS VF **(B)**, right putamen-PVS VF **(C)** with MoCA. Demographics and VRFs were regarded as covariates. Path coefficients with *p* values (*p < 0.05, **p < 0.01, ***p < 0.001, respectively). ALPS, analysis along the perivascular space; CSVD, cerebral small vessel disease; PVS, perivascular spaces; VF, volume fraction; VRFs, vascular risk factors.

In the mediation analysis, the ALPS index served as a significant partial mediator in the relationship between putamen-PVS VF and MoCA scores (mediation effect% = 25.2%; indirect effect = −40.720, 95% CI = −84.590 to −8.295; *p* < 0.05) (Figure 6A). Notably, when examining hemispheric contributions separately, the ALPS index partially mediated the association specifically for the left putamen PVS volume fraction (mediation effect% = 31.5%; indirect effect = −66.586, 95% CI = −138.105 to −4.833; *p* < 0.05) (Figure 6B), whereas no significant mediation effect was observed for the right putamen (Figure 6C).

## 4 Discussion

In this study, patients with CSVD exhibited a reduced ALPS index alongside an increased PVS volume fraction in the BG, caudate, and putamen. A notable left lateralization effect of PVS volume fraction was observed specifically within the BG and putamen of the CSVD group. Furthermore, a lower ALPS index was correlated with higher PVS volume fraction in these regions and poorer cognitive performance. Mediation analysis indicated that the ALPS index partially mediated the relationship between putamen PVS and MoCA scores, particularly in the left hemisphere.

The glymphatic system plays a vital role in maintaining brain homeostasis by facilitating waste clearance (Mestre et al., 2020). A decreased ALPS index is thought to reflect impaired glymphatic function and has been linked to several neurological disorders (Taoka et al., 2017; Hsu et al., 2023). Our results showed a significantly lower ALPS index in CSVD patients, with the most pronounced reduction in the CSVD-MCI subgroup, indicating a progressive decline in glymphatic function with disease severity. As a key anatomical component of the glymphatic system, PVS serves as an essential pathway for brain waste clearance (Iliff et al., 2012; Yu et al., 2022). Dysfunction of the glymphatic system may disrupt CSF–ISF exchange, leading to PVS enlargement (Yu et al., 2022). Previous studies have linked deep cerebral ePVS to aging and hypertension, supporting its role as a marker of underlying vascular pathology (Ding et al., 2017; Bouvy et al., 2016; Gyanwali et al., 2019; Charisis et al., 2023). Unlike semi-quantitative visual rating scales, our study employed a quantitative PVS volume fraction analysis, providing a more objective and precise assessment of PVS burden.

We found that an increased ALPS index was negatively correlated with BG-PVS volume fraction, underscoring an interplay between glymphatic function and PVS burden. Notably, while we observed a significant reduction in ALPS index in the CSVD-MCI subgroup, the corresponding increase in BG-PVS volume fraction did not reach statistical significance. This aligns with previous studies suggesting that glymphatic impairment may precede visible structural changes in PVS (Tian et al., 2023; Zhang et al., 2021). Such a temporal sequence supports the hypothesis that glymphatic impairment represents an early event in CSVD pathogenesis, potentially contributing to PVS dilation through compromised clearance along perivascular drainage pathways (Wardlaw et al., 2020).

Arterial pulsatility serves as a primary driver of glymphatic flow. Age-related arterial stiffening and hypertension can diminish CSF flow, contributing to PVS dilation (Sun et al., 2018; Naessens et al., 2020). The BG region appears particularly vulnerable to such disruptions as it receives substantial CSF influx via ventral perforating arteries, a characteristic that may underlie regional susceptibility to glymphatic dysfunction (Mestre et al., 2017). Decreased glymphatic clearance promotes the accumulation of metabolic waste, ultimately triggering neuronal injury and a cascade of pathological events (Aribisala et al., 2014), with cognitive impairment as the primary consequence (Nedergaard and Goldman, 2020; Wardlaw et al., 2020).

In line with previous studies (Tang et al., 2022; Tian et al., 2023; Zhang et al., 2021), the significant association between glymphatic impairment and deficits in executive function and processing speed, rather than memory or language, aligns with the proposed pathophysiology of CSVD. CSVD primarily affects subcortical-frontal networks, including the thalamo-cortical and striato-frontal circuits, which are critical for attention, processing speed, and executive control. Glymphatic dysfunction in deep white matter and basal ganglia regions may disproportionately disrupt the efficient communication within these widespread networks, leading to the characteristic “frontal-subcortical” cognitive profile. In contrast, memory and language functions rely more heavily on medial temporal and cortical regions, which may be affected at later stages of CSVD or through different mechanisms.

Notably, the association between the ALPS index and cognitive performance remained significant after adjustment for conventional neuroimaging markers, indicating its value as an independent indicator of cognitive decline. Mediation analysis further revealed that the ALPS index partially mediated the relationship between putamen PVS volume fraction and MoCA scores in CSVD patients, with a more pronounced effect observed in the left hemisphere. To our knowledge, this study is the first to report such a mediation effect, highlighting a potential pathway through which glymphatic dysfunction contributes to cognitive impairment in CSVD.

This regional specificity in cognitive deficits can be explained by the neuroanatomical and functional roles of the subcortical structures most affected in our cohort, particularly the putamen. The putamen, as a key component of the dorsal striatum, is integral to the fronto-striatal circuits that support executive functions and processing speed (Parent and Hazrati, 1995; Herrero et al., 2002). These circuits, which connect the putamen to the prefrontal cortex via the thalamus, are fundamental for higher-order cognitive processes, including executive control, planning, set-shifting, and the regulation of processing speed (Herrero et al., 2002; Schroeter, 2022). Pathology within the putamen, such as the waste accumulation suggested by enlarged PVS due to glymphatic dysfunction, would disrupt the delicate balance of these circuits, leading to the characteristic cognitive profile of CSVD (Wang J. et al., 2023; Wang M. et al., 2023; Schroeter, 2022). Our finding of a left-lateralized effect in the putamen is particularly noteworthy. The left hemisphere is dominant for motor planning and sequential processing in right-handed individuals, and prior studies have suggested that left putamen atrophy or dysfunction is more strongly associated with cognitive decline in neurodegenerative diseases such as Alzheimer’s disease and Parkinson’s disease (de Jong et al., 2008; Wylie et al., 2023). In vascular cognitive impairment, we also observed that cognitive deficits were associated with structural alterations in the left basal ganglia and left cortical regions (Liu et al., 2022; Huang et al., 2025). The widespread nature of glymphatic flow disruption likely impacts deep white matter tracts that connect various cortical areas, contributing to a more generalized cognitive slowing and inefficiency that underlies multiple cognitive domains. This provides a direct mechanistic explanation for why putamen-PVS volume, mediated by the ALPS index, was linked to global cognition deficits in our cohort. In contrast, the lack of a strong association with memory and language functions suggests that the glymphatic impairment in our CSVD cohort may not yet have significantly affected medial temporal lobe structures like the hippocampus (beyond PVS volume changes) or cortical language areas to the same extent, which is consistent with the early-to-mid stage of our patient population. It should be noted, however, that the interpretation of this lateralization effect remains preliminary due to the relatively small sample size in our study, and future validation in larger cohorts is warranted.

The thalamus, a pivotal hub in striatothalamo-cortical loops, plays a critical role in regulating both motor and cognitive processes (Herrero et al., 2002). Enlarged PVS in the thalamus has previously been associated with established neuroimaging markers of CSVD (Ding et al., 2017; Bouvy et al., 2016; Gyanwali et al., 2019; Charisis et al., 2023). In our study, we observed a negative correlation between the thalamus–PVS and ALPS index, which may be related to the thalamus’s role in connecting the putamen and the prefrontal cortex. It is noteworthy that in our study, hippocampal PVS volume was associated with visuospatial function but not with memory or language performance. This finding may reflect the specific role of the hippocampus in spatial navigation (Epstein et al., 2017). Furthermore, hippocampal PVS may serve as a biomarker of global CSVD burden, with its link to visuospatial dysfunction potentially arising from CSVD-related disruptions in prefrontal-subcortical circuits and widespread subcortical white matter injury (Wardlaw et al., 2020; Dichgans and Leys, 2017). The lack of association between the ALPS index and hippocampal PVS could reflect the typical phased and regional characteristics of CSVD pathology. Our cohort likely represents an early disease stage in which glymphatic dysfunction predominantly affects prefrontal-subcortical circuits while sparing medial temporal lobe structures (Herrero et al., 2002; Schroeter, 2022). Methodologically, the ALPS index, derived at the level of the lateral ventricles, may not adequately capture local glymphatic dynamics within the deeply situated and anatomically complex hippocampus (Iliff et al., 2012), which may be more strongly influenced by local vascular factors (van Veluw et al., 2020). Given the observational cross-sectional design and limited sample size of our study, these findings should be interpreted with caution and require validation in larger, more phenotypically diverse cohorts.

Although the correlations between the ALPS index and both PVS volume fraction and cognitive performance were statistically significant after FDR correction, it is important to note that the observed effect sizes were generally small to moderate. This is consistent with the multifactorial nature of cognitive impairment in CSVD, where glymphatic dysfunction is likely one of several contributing mechanisms, including vascular injury, neuroinflammation, and neurodegenerative pathology (Wardlaw et al., 2019; Nedergaard and Goldman, 2020). Furthermore, the DTI-ALPS index is an indirect measure, and its relationship with true glymphatic flow may be influenced by several confounding factors, including partial volume effects and regional anatomical variability. Future studies with larger samples, longitudinal designs, and more direct measures of glymphatic function are needed to better quantify the contribution of glymphatic impairment to CSVD progression and cognitive decline. Beyond statistical significance, our findings hold potential clinical implications for the management of CSVD. The ALPS index, as a non-invasive MRI biomarker, could serve several prospective roles. Within the CSVD spectrum, glymphatic dysfunction may represent a relatively early event. Consequently, the ALPS index could help identify CSVD patients at the highest risk for cognitive decline, enabling earlier, more preventive management strategies (Lu et al., 2025). Furthermore, given that glymphatic function is potentially modifiable, such as sleep modulation, the ALPS index could be developed as a quantitative tool to track disease progression or evaluate the efficacy of interventions aimed at enhancing waste clearance in the brain (Hablitz et al., 2019). Finally, our findings position that the glymphatic system plays a critical role in the mechanism of cognitive impairment in CSVD, providing a theoretical foundation for developing innovative treatments targeting the enhancement of glymphatic clearance. While these applications require validation in longitudinal and interventional studies, our study provides a foundational rationale for exploring the clinical utility of glymphatic imaging in CSVD.

Furthermore, recent studies have highlighted the critical role of sleep in glymphatic clearance, with evidence showing that glymphatic activity is significantly enhanced during slow-wave sleep (Hablitz et al., 2019; Fultz et al., 2019). Sleep disturbances, which are common in CSVD, may exacerbate glymphatic impairment and contribute to the accumulation of neurotoxic waste products. Our study did not include an assessment of sleep quality or patterns, which could be a significant confounding factor. Future studies that integrate polysomnography or detailed sleep questionnaires with the DTI-ALPS method will be crucial to elucidate the interplay between sleep, glymphatic clearance, and CSVD progression.

The strengths of this study are the quantification of the volume of PVS and the subdivision of the BG for the first time. Several limitations should also be considered. First, as a retrospective single-center study with a limited sample size, sample selection bias may have been introduced. Most importantly, we cannot infer causal relationships between glymphatic function and CSVD from our correlational data. Future studies with larger, multi-center cohorts, and longitudinal designs are warranted to establish causality. Second, manual ROI delineation in the calculation of the ALPS index may introduce potential bias, requiring automated delineation methods in future studies. Third, due to the smaller size and greater susceptibility to partial volume effects, reliable quantification of cortical PVS remains technically challenging with conventional MRI. Future studies with advanced imaging techniques capable of accurately quantifying cortical PVS are warranted to provide a more comprehensive picture. Finally, we did not systematically collect data on sleep quality, depressive symptoms, or medication use, known to influence glymphatic function and cognition, which may confound the relationships we observed. Future studies should include comprehensive assessments of these factors to better isolate the specific contributions of glymphatic dysfunction to CSVD-related cognitive impairment.

## 5 Conclusion

In conclusion, our findings demonstrate impaired glymphatic function in patients with CSVD, which correlates with worse cognitive performance. These results suggest that the DTI-ALPS index may serve as a promising neuroimaging biomarker, potentially aiding in the identification of CSVD patients with cognitive impairment and providing insights into the underlying pathophysiology of the disease.

## Data Availability

The original contributions presented in the study are included in the article/Supplementary material, further inquiries can be directed to the corresponding author.

## Footnotes

^1^ https://www.processmacro.org/

## Supplementary material

SUPPLEMENTARY TABLE S1 Detailed results of ALPS index and PVS volume fraction across all analyzed brain regions.

SUPPLEMENTARY TABLE S2 Complete results of partial correlation analyses between ALPS index and PVS volume fraction in CSVD group.

SUPPLEMENTARY TABLE S3 Complete results of partial correlation analyses between ALPS index and cognitive function in CSVD group.
