# Supplementary material for: Glymphatic system impairment in cerebral small vessel disease: associations with perivascular space volume and cognition
Source: Front Aging Neurosci. 2025 Nov 13;17:1680094. doi: 10.3389/fnagi.2025.1680094 (PMC12657371; doi:10.3389/fnagi.2025.1680094)
Supplement: SUPPLEMENTARY TABLE S1 — Detailed results of ALPS index and PVS volume fraction across all analyzed brain regions. [file Table_1.DOCX]

**Supplementary Information**

**Glymphatic system impairment in cerebral small vessel disease: associations with perivascular space volume and cognition**

**Supplementary Tables**

Table S1 ALPS index and subcortical nucleus PVS VF among HC, CSVD-NCI, and CSVD-MCI groups

|  | HC  (n=40) | CSVD-NCI  (n=52) | CSVD-MCI  (n=68) | *p*-value | Post-hoc analysis | | |
| --- | --- | --- | --- | --- | --- | --- | --- |
|  |  |  |  |  | HC vs.  CSVD-NCI | HC vs.  CSVD-MCI | CSVD-NCI vs.  CSVD-MCI |
| ALPS index | 1.39 ± 0.14 | 1.29 ± 0.11 | 1.23 ± 0.11 | <0.001^***^ | <0.001^***^ | <0.001^***^ | 0.0048^*^ |
| Subcortical nucleus PVS |  |  |  |  |  |  |  |
| Thalamus-PVS VF (%) | 0.0661 ± 0.0080 | 0.0705 ± 0.0095 | 0.0683 ± 0.0098 | 0.0838 |  |  |  |
| Hippocampus-PVS VF (%) | 0.0111 ± 0.0038 | 0.0125 ± 0.0041 | 0.0141 ± 0.0048 | 0.0021^**^ | 0.1089 | 0.0009^**^ | 0.0457 |
| BG-PVS VF (%) | 0.0632 ± 0.0090 | 0.0718 ± 0.0059 | 0.0714 ± 0.0103 | <0.001^***^ | <0.001^***^ | <0.001^***^ | 0.7788 |
| Amygdala-PVS VF | 0.0044 ± 0.0010 | 0.0048 ± 0.0013 | 0.0048 ± 0.0015 | 0.2415 |  |  |  |
| Caudate-PVS VF | 0.0189 ± 0.0038 | 0.0226 ± 0.0034 | 0.0213 ± 0.0041 | <0.001^***^ | <0.001^***^ | 0.0035^*^ | 0.0650 |
| Pallidum-PVS VF | 0.0107 ± 0.0035 | 0.0107 ± 0.0032 | 0.0103 ± 0.0032 | 0.7036 |  |  |  |
| Putamen-PVS VF | 0.0219 ± 0.0084 | 0.0260 ± 0.0060 | 0.0274 ± 0.0073 | <0.001^***^ | 0.0106^*^ | 0.0005^**^ | 0.2613 |

Note: Data are presented as mean ± standard deviation. Group comparisons for normally distributed variables were performed using one-way analysis of variance (ANOVA) with post-hoc Bonferroni correction for pairwise comparisons. Abbreviations: HC, healthy control; CSVD, cerebral small vessel disease; NCI, no cognitive impairment; MCI, mild cognitive impairment; ALPS, analysis along the perivascular space; PVS, perivascular space; VF, volume fraction; BG, basal ganglia.

PVS VF = PVS volume / (gray+ white matter volume) *100%

**p* < 0.05; ***p* < 0.01; ****p* < 0.001 under Bonferroni correction

Table S2 Partial correlation analysis of ALPS index with neuroimaging markers in CSVD group

| variable | Model 1 | |  | Model 2 | |
| --- | --- | --- | --- | --- | --- |
|  | r | p |  | r | p |
| WMH Fazekas scores | -0.280 | 0.002* |  | -0.231 | 0.014* |
| Lacunes | -0.109 | 0.241 |  | -0.065 | 0.497 |
| CMBs | -0.195 | 0.036 |  | -0.176 | 0.063 |
| BG-ePVS | -0.597 | <0.001* |  | -0.663 | <0.001* |
| CSVD total burden | -0.459 | <0.001* |  | -0.467 | <0.001* |
| Hippocampus-PVS VF | -0.142 | 0.126 |  | -0.152 | 0.110 |
| Thalamus-PVS VF | -0.214 | 0.020* |  | -0.213 | 0.024* |
| BG-PVS VF | -0.234 | 0.011* |  | -0.232 | 0.014* |
| Amygdala-PVS VF | -0.099 | 0.290 |  | -0.067 | 0.480 |
| Caudate-PVS VF | -0.206 | 0.026* |  | -0.221 | 0.019* |
| Pallidum-PVS VF | 0.084 | 0.367 |  | 0.089 | 0.350 |
| Putamen-PVS VF | -0.214 | 0.021* |  | -0.210 | 0.026* |

Note: Partial correlation analyses were performed to control for potential confounders. Model 1 was adjusted for age, sex, and years of education. Model 2 was further adjusted for vascular risk factors (VRFs), including hypertension, diabetes, hypercholesterolemia, smoking, and BMI. The statistical significance of correlation coefficients (r) was assessed, and p-values were corrected for multiple comparisons using the false discovery rate (FDR) method. Abbreviations: ALPS, analysis along the perivascular space; CSVD, cerebral small vessel disease; WMH, white matter hyperintensity; CMBs, microbleeds; BG, basal ganglia; ePVS, enlarged perivascular space; VF, volume fraction.

**p* <0.05 after FDR correction.

Table S3 Partial correlation analysis of ALPS index with cognition in CSVD group

| variable | Model 1 | |  | Model 2 | |  | Model 3 | |
| --- | --- | --- | --- | --- | --- | --- | --- | --- |
|  | r | p |  | r | p |  | r | p |
| Global cognitive function | 0.386 | <0.001* |  | 0.385 | <0.001* |  | 0.312 | 0.001* |
| Memory function | -0.002 | 0.986 |  | -0.020 | 0.834 |  | -0.033 | 0.737 |
| Executive function | 0.253 | 0.006* |  | 0.258 | 0.006* |  | 0.242 | 0.012* |
| Processing speed | 0.300 | 0.001* |  | 0.294 | 0.002* |  | 0.264 | 0.006* |
| Language function | 0.066 | 0.478 |  | 0.053 | 0.579 |  | 0.088 | 0.367 |
| Visuospatial function | 0.363 | <0.001* |  | 0.360 | <0.001* |  | 0.272 | 0.004* |

Note: Partial correlation analyses were conducted to control for potential confounders. Model 1 was adjusted for age, sex, and years of education. Model 2 was further adjusted for vascular risk factors (VRFs), including hypertension, diabetes, hypercholesterolemia, smoking, and BMI. Model 3 was additionally adjusted for conventional CSVD neuroimaging markers, including WMH Fazekas scores, the presence of lacunes, the presence of CMBs, and BG-ePVS grade. The statistical significance of correlation coefficients (r) was assessed, and p-values were corrected for multiple comparisons using the false discovery rate (FDR) method. Abbreviations: ALPS, analysis along the perivascular space; CSVD, cerebral small vessel disease.

**p* <0.05 after FDR correction.
